# The Dual Roles of Esophageal Submucosal Glands: Protection and Pathogenesis in Benign and Malignant Disease: A Scoping Review

**DOI:** 10.3390/cells15191727

**Published:** 2026-09-22

**Authors:** Chun-Ting Chen, Michiel F. G. de Maat, Ann Driessen, Michael Doukas, Bas P. L. Wijnhoven, Maikel P. Peppelenbosch, Kausilia K. Krishnadath, Vincent T. Janmaat

**Affiliations:** 1 Department of Gastroenterology and Hepatology, University Medical Centre Rotterdam (Erasmus MC), Erasmus University Rotterdam, Dr. Molewaterplein 40, 3015 GD Rotterdam, The Netherlands; c.chen@erasmusmc.nl (C.-T.C.);; 2 The Laboratory of Experimental Medicine and Pediatrics (LEMP), Antwerp University, Universiteitsplein 1, Wilrijk, 2610 Antwerp, Belgium; 3 Department of Abdominal Surgery/eSophageal Oncology Network Antwerp Region (SONAR), University Hospital Antwerp (UZA), Antwerp University, Drie Eikenstraat 655, Edegem, 2650 Antwerp, Belgium; 4 Department of Pathology, University Hospital Antwerp (UZA), Antwerp University, Drie Eikenstraat 655, Edegem, 2650 Antwerp, Belgium; 5 Department of Pathology, University Medical Centre Rotterdam (Erasmus MC), Erasmus University Rotterdam, Dr. Molewaterplein 40, Erasmus MC, 3015 GD Rotterdam, The Netherlands; 6 Department of Surgery, University Medical Centre Rotterdam (Erasmus MC), Erasmus University Rotterdam, Dr. Molewaterplein 40, Erasmus MC, 3015 GD Rotterdam, The Netherlands; 7 Department of Gastroenterology and Hepatology, University Hospital Antwerp (UZA), Antwerp University, Drie Eikenstraat 655, Edegem, 2650 Antwerp, Belgium

**Keywords:** esophageal submucosal glands, Barrett’s esophagus, gastroesophageal reflux disease, epithelial plasticity, esophageal adenocarcinoma, scoping review

## Abstract

**Background**: Esophageal submucosal glands (SMGs) contribute to mucosal defense through bicarbonate, mucin, and reparative-factor secretion. Chronic reflux may impair these functions and remodel the glandular compartment, potentially linking SMG injury to reflux esophagitis, Barrett’s esophagus (BE), and esophageal adenocarcinoma (EAC). **Objective:** To map the human, animal, and in vitro literature on the physiological and pathological roles of esophageal SMGs and to identify evidence gaps relevant to reflux injury, BE, EAC, and endoscopic therapy. **Methods:** This scoping review was reported in accordance with PRISMA-ScR. Embase, Web of Science, PubMed, and Google Scholar were searched from inception to 15 January 2025. Two reviewers independently screened records against prespecified eligibility criteria. The protocol was not registered. **Results:** The review included 97 full-text publications and 7 conference abstracts. The full-text set comprised 89 original studies and 8 reviews; review articles were used for context and citation tracing rather than counted as independent primary evidence. The mapped literature supports secretory and reparative functions of SMGs and documents injury-associated remodeling, including loss of mucinous acini and expansion of atrophic, oncocytic, or duct-like phenotypes. Marker expression, spatial continuity, and clonal observations are compatible with glandular participation in repair and Barrett-associated biology, but do not establish a unique cell of origin and lineage relationships remain unresolved. **Conclusions:** Esophageal SMGs occupy a plausible interface between mucosal protection and pathological plasticity. The most consequential gaps are the lack of longitudinal human evidence, source-resolved lineage information, standardized reporting of glandular phenotypes, and human models that reproduce reflux exposure and the stromal niche.

## 1. Introduction

Esophageal submucosal glands (SMGs) were historically compared with minor salivary glands because of their morphology and secretory profile. They are now regarded as specialized esophageal compartments with local defensive and regenerative functions. SMGs exhibit different patterns of location among species. In human esophagus, they are enriched in the proximal and distal third areas, including the gastroesophageal junction (GEJ) [1,2].

SMGs release bicarbonate, mucins, epidermal growth factor (EGF), prostaglandin E2 (PGE2), and other mediators into the esophageal lumen [3,4,5,6]. These contribute to pre-epithelial buffering, lubrication, and repair after reflux-associated injury.

The same compartment also undergoes substantial remodeling in disease. Persistent acid and bile exposure has been associated with impaired secretion, inflammation, acinar loss, ductal expansion, and metaplastic phenotypes. These observations have prompted the hypothesis that altered SMG or ductal populations may contribute to Barrett’s esophagus (BE) and esophageal adenocarcinoma (EAC) [7,8,9]. Most evidence is spatial, phenotypic, or retrospective, however, and does not identify an exclusive cellular origin.

BE is characterized by the replacement of stratified squamous epithelium with metaplastic columnar epithelium and is the recognized precursor lesion for EAC [10]. Proposed sources of Barrett epithelium include surface squamous progenitors, junctional epithelial populations, gastric cardia cells, and deeper glandular compartments. Rare SMG-associated neoplasms, including mucoepidermoid carcinoma and duct adenoma or adenocarcinoma, further demonstrate that glandular epithelial populations can acquire neoplastic phenotypes distinct from conventional Barrett-associated EAC [11].

The evidence base is difficult to synthesize because it spans descriptive human histopathology, physiology, case reports, conference abstracts, large-animal injury models, rodent junctional models, and in vitro systems. Standard rodents lack esophageal SMGs, and viable human SMG models remain scarce. Consequently, several issues remain unsettled, including the existence and lineage potential of glandular progenitors, the relationship between acinar–ductal metaplasia and Barrett-like epithelium, and the contribution of SMGs to repair after ablative therapy. This breadth and heterogeneity favor an evidence-mapping approach over quantitative effect estimation.

Therefore, this scoping review poses the following question: what is known about the physiological and pathological roles of esophageal SMGs across human, animal, and in vitro evidence, and which gaps prevent causal and translational conclusions? This study employs a population–concept–context framework, with data sources covering human tissues and relevant comparative models (population); SMG structure, secretion, injury, repair, remodeling, and neoplasia (concept); and healthy, reflux-associated, metaplastic, malignant, and post-treatment settings (context).

This study aims to map the scope and characteristics of the relevant literature by systematic search methods, including summarizing evidence related to mucosal protection, reflux injury, BE and EAC, rare glandular neoplasms, and endoscopic treatment as well as distinguishing primary observations from secondary interpretation and identifying priorities for mechanistic investigation.

## 2. Methods

### 2.1. Study Design and PRISMA Guidelines

This scoping review was conducted to map a heterogeneous body of evidence rather than estimate a pooled intervention effect. Reporting follows the Preferred Reporting Items for Systematic Reviews and Meta-Analyses extension for Scoping Reviews (PRISMA-ScR) [12]. No protocol was published or prospectively registered.

### 2.2. Information Sources and Search Strategy

EMBASE, Web of Science, PubMed, and Google Scholar were searched from database inception to 15 January 2025. Controlled vocabulary and free-text terms addressed esophageal SMGs, BE, EAC, protective mechanisms, reflux, mucin secretion, and glandular metaplasia. Embase-indexed conference abstracts from 1985 to 2024 were also screened to capture evidence not yet available as a full article. Complete source-specific search strings, limits, and retrieval counts are provided in Supplementary Materials.

### 2.3. Eligibility Criteria

Eligibility criteria were defined before screening to support transparent source selection (Figure 1).

Eligible sources directly evaluated the anatomy, physiology, pathology, molecular characteristics, or treatment-associated responses of esophageal SMGs. Human studies and relevant animal or in vitro models published in English were included. Original articles and conference abstracts contributed to the primary findings. Review articles were retained only to map existing interpretations, clarify terminology, and trace citations; they were not treated as independent confirmation of primary observations.

Sources were excluded when they focused exclusively on other glandular structures, mentioned esophageal SMGs without extractable evidence or additional insight, duplicated data reported in an included peer-reviewed article, or were not published in English. Conference abstracts were excluded when the same dataset was available in a full publication.

### 2.4. Study Selection and Data Extraction

All records were exported to reference-management software and deduplicated. Two reviewers independently screened titles and abstracts and then assessed full texts against the eligibility criteria. Disagreements were resolved by discussion and consensus, with consultation of a senior investigator when required. Data were charted in a structured matrix covering publication year, source type, species and model, population and reported sample size, tissue or disease context, SMG compartment, principal observations, and study-level limitations.

### 2.5. Critical Appraisal and Certainty Assessment

A formal risk-of-bias or critical-appraisal tool was not applied. This decision reflects the scoping objective and the inclusion of highly heterogeneous source types, including descriptive histology, physiology, case reports, conference abstracts, animal models, and in vitro studies. Instead, a narrative-style critical appraisal was integrated into the text to evaluate the literature. This approach aligns with PRISMA-ScR guidelines, which treats critical appraisal as optional when it is not the primary objective of a scoping review [12]. Consequently, this review maps reported evidence and its limitations but does not rank study validity, estimate certainty, or infer pooled effects.

### 2.6. Evidence Synthesis

Charted information was organized by species or model and categorized by subject area, including physiology, reflux-associated injury and repair, BE and EAC, rare glandular neoplasms, and treatment-associated outcomes. Findings were synthesized descriptively. Review articles were used to contextualize terminology and identify primary reports but were not counted as independent confirmation. Quantitative meta-analysis was not planned because the review objective was evidence mapping and the outcomes, populations, and designs were not sufficiently comparable.

## 3. Results

The searches identified 3191 records, including 242 Embase-indexed conference abstracts. After removal of 1926 duplicates, 1265 unique records underwent title and abstract screening, and 1130 were excluded. One hundred and thirty-five reports were assessed for eligibility: one hundred and twenty-eight full-text publications and seven conference abstracts. Thirty-one full-text reports were excluded because they did not address esophageal SMGs (*n* = 13), focused on Brunner’s glands (*n* = 12), mentioned SMGs without extractable evidence (*n* = 3), were theses duplicating published data (*n* = 2), or were not in English (*n* = 1). The final map contained 104 sources: 89 original full-text studies, 8 review articles used for context, and 7 conference abstracts (Figure 1).

### 3.1. Physiology

The included evidence covered human observational and physiological studies, comparative mammalian models, and in vitro culture systems. Species-specific anatomy materially affects interpretation because commonly used rodents lack SMGs, whereas humans, pigs, dogs, and opossums possess glandular structures with different degrees of physiological comparability (Table 1). Source-level characteristics are summarized in Supplementary Table S1.

#### 3.1.1. Human Data

Human SMGs are distributed throughout the esophagus and are enriched proximally and distally, with high density near the gastroesophageal junction [5,15,18,19,20]. Human and porcine esophagi share a non-keratinized stratified squamous lining and contain SMGs that secrete mucus, bicarbonate, and growth factors [5,13,14,25,26]. Commonly used rodents have a keratinized squamous lining and lack esophageal SMGs. This contrast may reflect species-specific reliance on mechanical versus secretory protection [36]. Each human gland contains deep submucosal acini and excretory ducts that traverse the muscularis mucosae and lamina propria to reach the epithelial surface [25,37].

Histological and molecular markers distinguish SMG compartments. NY-ESO-1 has been detected in glandular units and proposed as a marker of repair-associated cells [17], whereas p63 marks basal epithelial populations and is required for normal squamous stratification [34]. Laminin alpha1 localizes to the basement membrane of SMGs and their ducts but is largely absent from surface squamous epithelium [16]. Functional studies identify duct cells and serous demilunes as contributors to alkaline secretion, which is stimulated by muscarinic signaling and involves sodium–bicarbonate cotransport and chloride–bicarbonate exchange [13,14,30,31,38,39].

SMGs also produce mucins, particularly MUC5B, and growth factors such as EGF [4,40]. Mucin expression varies with epithelial compartment and differentiation: MUC1 and MUC4 are associated with surface squamous epithelium, whereas MUC5B characterizes glandular acini [4,40]. TFF3 peptide and transcript have been localized to human esophageal SMGs but not the overlying stratified squamous epithelium [41]. Secreted mucins form a gel-like layer that retains bicarbonate, and human perfusion studies report increased mucin secretion after acid or acid–pepsin exposure [42].

Secretory output changes during mucosal injury and repair. Studies of reflux disease and healing reported altered releases of mucins, EGF, and PGE2 [43,44]. Reflux esophagitis may impair both esophageal and salivary gland secretion, illustrating the integration of these protective systems [44]. A conference abstract further reported sex-associated differences in the secretory response to serotonergic stimulation in patients with GERD. Females showed larger increases in protective protein secretion, whereas males exhibited greater bicarbonate secretion [45].

#### 3.1.2. Animal Data

Porcine and opossum studies have defined complementary features of SMG composition and function [21,23,33]. Porcine glands contain p63-positive basal populations and CK7-positive glandular or ductal populations. Isolating those glands can generate spheroids with distinct squamous-like and duct-like phenotypes [21,23,25]. These observations demonstrate phenotypic heterogeneity and injury responsiveness, but do not establish biopotency of an individual cell.

Experimental activation of the ClC-2 chloride channel reduces acid-associated injury in porcine tissue [24]. In perfused porcine esophagus, basal alkali secretion was predominantly bicarbonate; carbachol increased this response, whereas atropine and carbonic-anhydrase inhibition reduced it [14]. The 5-HT4 agonist tegaserod increased bicarbonate output in porcine preparations [22,46].

Opossum and rabbit comparisons also support an SMG contribution to luminal neutralization [30,31,32,33]. Opossums, which possess a dense glandular network, show greater alkaline secretion and acid responsiveness than rabbits.

Across these models, SMG secretion contributes to a lumen-to-surface pH gradient, although this buffering capacity is finite and may be exceeded in severely acidic environments where the pH falls below 2.0 [22,30].

### 3.2. Pathology

Reflux esophagitis and exocrine dysfunction.

#### 3.2.1. Human Data

SMG-associated ducts are spatially linked to squamous regeneration after acid suppression and after radiofrequency ablation (RFA) for BE [23,47,48,49]. Squamous islands may remain contiguous with underlying ducts, but wound-edge migration and other epithelial sources also contribute. Human kinetic studies further suggest that metaplastic columnar epithelium can proliferate more rapidly than native squamous epithelium [50]. In esophageal intramural pseudo diverticulosis, the characteristic outpouchings arise from chronically inflamed and dilated SMG ducts [51].

#### 3.2.2. Animal Data

Canine injury models show that deep gland-associated cells can persist after extensive surface-mucosal loss [28]. Repair varies with the reflux environment: persistent exposure favors columnar re-epithelialization, whereas acid suppression permits mixed repair containing squamous islands [28]. Reflux also induces proliferation in SMGs and ducts [29]. With sustained injury, glands may lose a mucinous phenotype and acquire atrophic or eosinophilic changes that express CK7 and SOX9 [52,53,54].

Bile-acid exposure produces transitional phenotypes in experimental systems. In mice, glycine-conjugated bile acids induce multilayered glands at the squamocolumnar junction, with co-expression of squamous and columnar markers and focal crypt fission [35]. In porcine SMG cultures, deoxycholic acid promotes columnar or glandular differentiation [55]. Because mice lack esophageal SMGs and the porcine observations arise from an ex vivo model, these findings demonstrate injury-associated epithelial plasticity rather than direct equivalence to human BE.

### 3.3. Barrett’s Esophagus and EAC

#### 3.3.1. Human Data

Chronic injury is associated with necrotizing sialometaplasia-like change (NSMLC) and oncocytic glandular metaplasia (OGM) in human SMGs [52,54]. Both patterns involve loss of mucinous acini and replacement by squamoid, duct-like, or oncocytic cells, often with reduced MUC5B. These changes are compatible with impaired glandular defense, although the direction of causality between gland injury and surface disease is unresolved.

Comparisons across normal esophagus, high-grade dysplasia, and EAC identify progressive abnormalities within the SMG compartment [52,53]. Acinar–ductal metaplasia (ADM) and OGM appear to occupy an injury-remodeling spectrum, whereas NSMLC may represent a distinct ischemic or inflammatory response. Glandular abnormalities often parallel changes in the overlying epithelium, but spatial concordance alone does not establish lineage.

Barrett-associated neoplasia is accompanied by distorted glandular architecture, fewer mucinous acini, and more atrophic or inflamed acini beneath abnormal epithelium [18,52,53]. Acid exposure has also been reported to increase gastrin-receptor expression in SMGs [56]. Whether this signaling contributes directly to human disease progression remains untested (Figure 2).

Several studies have identified progenitor-associated phenotypes within SMGs and their excretory ducts [53,57]. Atrophic or oncocytic acini may express CK5, CK6, CK7, SOX2, and SOX9, consistent with epithelial plasticity or an altered differentiation state. Single-cell comparisons have also identified SMG-associated populations with transcriptional similarity to BE [57]. These data nominate glandular populations for mechanistic study but do not demonstrate that SMGs are the exclusive origin of BE [58].

The EphB2-MYC pathway provides one experimentally investigated link between epithelial state and Barrett-associated programs [59]. EphB2 and MYC were induced in SMGs with ADM and in porcine BE-like SMG spheroids; pathway perturbation in other esophageal models affected proliferation and differentiation. The causal sequence within human SMGs, however, has not been established.

BE displays mixed gastric and intestinal differentiation, including MUC5AC, MUC6, and, in intestinalized lesions, MUC2 [4,40]. Hybrid epithelia near the neo-squamocolumnar junction can co-express the squamous marker CK13 and the columnar markers CK7 and MUC5AC [60]. CK7-positive duct-associated cells have also been observed within BE segments [61]. These phenotypes overlap with SMG ducts but do not define a unique developmental route [62].

Three-dimensional histological reconstruction has shown continuity between squamous islands and native glandular ducts [63]. SMGs cluster near the squamocolumnar junction in columnar-lined esophagus (CLE) [64], and glands beneath columnar epithelium may be hyperplastic [64]. Population-based histology also identifies SMGs and ducts among features associated with CLE [65]. These observations support local participation in repair while remaining compatible with field injury, selection, or expansion of pre-existing populations.

SMG-associated phenotypes vary across disease stages [54,66]. Increasing epithelial heterogeneity during Barrett progression can support expansion of selected clones [66]. Laser-capture studies indicate that mutant Barrett clones may expand through crypt fission rather than solely by lateral spread [67], but these analyses cannot retrospectively reconstruct every earlier cell-state transition.

Naturally occurring mitochondrial and nuclear markers have been used to assess clonal organization. Cytochrome-c-oxidase-deficient patches occur in normal squamous epithelium and individual Barrett glands. Such lineage marks indicate that Barrett glands are clonal structures maintained by multiple long-lived stem cells with multilineage potential [68]. They do not, however, uniquely identify the anatomical source of the initiating cell.

Evidence concerning regenerated squamous epithelium is mixed. Some squamous islands share clonal alterations with adjacent Barrett glands, supporting a local common ancestor [20,47]. In other patients, TP53 and CDKN2A patterns suggested that neosquamous epithelium arose from a lineage distinct from BE [69]. Leedham et al. identified a p16 alteration in a superficial SMG duct and adjacent Barrett crypts, whereas nearby neosquamous islands were wild type [67]. Together, these findings are compatible with multiple local progenitor populations and do not distinguish reprogramming from selection in every case.

Progressive glandular injury may reduce local reflux defense and create a permissive environment for metaplasia [66]. BE-to-EAC progression is associated with accumulating genetic and chromosomal abnormalities [8,66,70,71], some of which have also been detected in gland-associated compartments [18,67]. It remains uncertain whether these alterations precede surface metaplasia or arise secondarily within a shared disease field.

Regenerated squamous epithelium may retain alterations present in its source population, potentially creating fields with continued neoplastic susceptibility [48,72,73]. CAV3 has been linked to SMG-associated epithelial homeostasis and repair [74]. Normal SMG epithelial cells express HLA-ABC, whereas reduced HLA-ABC expression in esophageal carcinoma may contribute to immune evasion [75]. Chromatin analyses further identify mixed gastrointestinal states in BE that overlap with selected gland-associated phenotypes but remain distinct from normal gastric tissue [76].

Spatial sampling and genetic reconstruction across BE segments provide evidence that columnar epithelium lacking goblet cells can carry premalignant clones [77]. Thus, neoplastic evolution may begin before a fully intestinalized phenotype is present.

Single-cell studies offer complementary but partly divergent models of Barrett epithelial identity. One atlas described a shared gastric-to-intestinal transition across esophageal and gastric metaplasia [78]. By contrast, a study comparing BE with adjacent esophagus, stomach, and duodenum identified LEFTY1- and OLFM4-marked Barrett populations with transcriptional similarity to SMGs [57]. Differences in tissue location, disease stage, reference populations, and exposure to the surrounding disease field may explain part of this divergence. These explanations remain hypotheses rather than resolved mechanisms.

#### 3.3.2. Animal Data

Animal and culture models further demonstrate esophageal epithelial plasticity. Porcine SMG cells form three-dimensional spheroids containing p63-positive and CK7-positive populations [23,25]. Single-cell analysis of porcine spheroids identified squamous-like and BE-like states and suggested higher EphB2-MYC pathway activity in the latter [59]. In mice, TP63 ablation disrupts normal squamous development [34], and chronic glycine-conjugated bile-acid exposure induces multilayered glandular changes at the squamocolumnar junction [35]. Because mice lack esophageal SMGs, these experiments model junctional plasticity rather than gland-derived human BE.

### 3.4. Mucoepidermoid Carcinoma and SMG Duct Adenoma

#### Human Data

SMGs are also implicated in rare neoplasms distinct from conventional EAC. Esophageal mucoepidermoid carcinoma (MEC) contains mucus-producing, intermediate, and epidermoid cells and is generally interpreted as SMG-associated [11,79,80]. In basaloid squamous cell carcinoma, SMG differentiation has been described in a subset of tumors [81]. These histological associations support a glandular relationship but do not by themselves distinguish direct clonal origin from differentiation within a local neoplastic field.

Benign and malignant tumors of SMG ducts are uncommon. Esophageal SMG duct adenomas occur predominantly in SMG-rich regions [82,83,84,85]. Reflux-associated pressure and inflammation have been proposed as proliferative stimuli, but this remains hypothetical [83]. A reported duct adenocarcinoma harbored KRAS, TP53, and SMAD4 alterations with regional loss of heterozygosity [86], suggesting a molecular route that may differ from conventional Barrett-associated EAC. Table 2 summarizes the mapped pathological settings and the evidential limits of each proposed SMG role.

### 3.5. SMGs in a Therapeutic Context

#### 3.5.1. Human Data

Early esophageal squamous neoplasia can extend into deeper wall layers along SMG ducts [49,90,94,97], potentially leaving disease beyond the reach of superficial ablation. Case reports also describe SMG-associated adenomas or adenocarcinomas presenting as submucosal lesions [79,80,81,83,84,85,86,91,92,93,94,95,96,97]. These observations support careful assessment of lesion depth and histological margins. They do not, however, define a validated EUS depth threshold or a universal rule favoring endoscopic submucosal dissection over RFA; modality selection should remain lesion-specific and multidisciplinary.

#### 3.5.2. Animal Data

In pigs, focal RFA and cryoablation can injure or ablate SMGs, whereas cooled RFA has a less deep effect [49]. Glands surviving surface ablation show increased Ki67 and SOX9 expression [23]. Alterations shared by treated surface mucosa and deep glands have been associated with recurrent dysplasia [98]. In a canine model of circumferential endoscopic submucosal dissection, healed segments lacked muscularis mucosae and SMGs and were replaced by collagen-rich granulation tissue [27]. These findings suggest that treatment depth may influence whether gland-associated repair is retained or replaced by fibrotic healing.

## 4. Discussion

This scoping review maps a context-dependent role for esophageal SMGs in secretion, repair, remodeling, and neoplasia. The strongest direct evidence concerns bicarbonate and mucin secretion, anatomical responses to injury, and histological associations with repaired or metaplastic epithelium. Claims concerning cellular origin or causal disease progression are substantially less secure because most studies are observational, retrospective, or performed in non-human systems.

In the healthy esophagus, SMGs contribute to mucosal defense through bicarbonate [14], mucins [4,40,41,99], and growth-factor secretion [56]. Their distal enrichment is relevant to reflux exposure, although severe reflux can affect the full organ. Salivary and esophageal secretions are functionally integrated [44]. PGE2 illustrates the context dependence of glandular mediators: basal secretion supports mucosal protection, whereas sustained elevation in BE has been associated with neoplastic signaling [100]. Aspirin-based chemoprevention has therefore been studied [101], but potential benefits must be weighed against bleeding risk and possible effects on acute repair.

Sex-associated differences provide a testable, but currently underdeveloped, biological question. Limited physiological evidence suggests that males and females may rely differently on bicarbonate and protective-protein secretion [45]. A plausible hypothesis is that sex-dependent glandular defense modifies cumulative mucosal injury and thereby contributes to the marked male predominance of EAC. Existing studies are too small and heterogeneous to support this link. Future work should therefore report sex as biological metadata, quantify gland composition and secretion, and test interaction between sex, reflux exposure, and epithelial outcome rather than treating sex as a post hoc subgroup.

Acid suppression promotes surface healing and is accompanied by altered mucin, EGF, and PGE2 secretion [43,44,45]. Direct evidence that proton-pump inhibitor therapy restores normal SMG architecture or sustained secretory capacity is limited. It is also unknown whether severely remodeled acini in OGM or NSMLC can revert to a protective phenotype, whether surface repair stabilizes the underlying glandular compartment, or whether abnormal ductal populations persist despite symptom control. Longitudinal biopsies or paired experimental models are required to distinguish functional recovery from surface re-epithelialization alone.

SMG dysfunction occurs within a wider anatomical context. Hiatal hernia compromises the antireflux barrier and can increase reflux burden [102,103]. Age and body mass index are associated with hiatal hernia, GERD, and EAC risk, but a direct longitudinal link between junctional failure, declining glandular buffering, and SMG remodeling has not been demonstrated.

With sustained injury, SMGs may shift from a protective compartment toward altered epithelial states. Histological continuity with ducts, glandular enrichment near metaplastic interfaces, and shared clonal alterations support local participation [20,47,57,63,67,68,69,88]. These findings remain compatible with at least three explanations: direct reprogramming of gland-associated cells, selection and expansion of a pre-existing local clone, or parallel changes driven by a shared injury field. None excludes contributions from surface, junctional, or gastric-cardia populations [60,104,105,106,107,108]. Single-cell studies illustrate this uncertainty. A study sampling SMGs adjacent to Barrett segments reported strong transcriptional similarity between SMG-associated and Barrett populations [57], whereas an atlas using other reference tissues supported a gastric-to-intestinal metaplastic program [8]. Differences in sampling location and disease-field exposure may contribute to the discrepancy, but this explanation remains a testable hypothesis rather than an established mechanism. Accordingly, SMG involvement should be described as a supported candidate mechanism, not as a proven universal cell of origin.

The therapeutic evidence is similarly suggestive rather than prescriptive. Duct-associated squamous islands may contribute to re-epithelialization after RFA, whereas extensive injury or gland loss may favor fibrosis [27,48]. Conversely, intraductal extension of squamous neoplasia can evade superficial treatment [49,90,94,97]. These observations justify careful pre-treatment staging and complete pathological assessment when ductal or deep extension is suspected. However, the literature does not support a universal EUS depth cutoff or treatment algorithm based solely on SMG density.

A useful experimental sequence should first establish tractable human SMG models and then add complexity only when it addresses a defined causal question. Patient-derived three-dimensional SMG cultures can test self-renewal and glandular differentiation, whereas two-dimensional or air–liquid-interface derivatives provide controlled access to the apical surface for acid and bile-acid exposure. Matched comparison with native SMG, squamous, gastric cardia tissues, and Barrett tissues, together with time-resolved phenotyping, would distinguish transient stress responses from stable epithelial-state changes [109]. Comparative porcine models remain valuable for gland physiology and wound depth, while mouse junctional models address epithelial plasticity but cannot directly model an esophageal SMG lineage.

Microfluidic systems could next incorporate luminal flow, intermittent reflux-like exposure, extracellular matrix, fibroblasts, and immune components. Human Barrett organ-chip studies have already shown that epithelial–stromal interactions can be modeled under compartmentalized flow [110]. An SMG-focused adaptation would permit separate measurement of luminal secretion, barrier function, ductal migration, and stromal signaling, while retaining donor-specific context. Such models should be benchmarked against native tissue rather than assumed to reproduce gland physiology by morphology alone.

Finally, lineage-informative experiments should combine clonal starting conditions with heritable recording and endpoint cell-state analysis. CRISPR-based barcoding can couple lineage history with single-cell and spatial transcriptomics in organoid systems [111], although its feasibility, editing effects, and barcode dropout must be validated specifically in primary SMG cultures. Used together, organoids, microfluidics, genetic perturbation, and spatial profiling could test whether reflux selects pre-existing glandular states or induces reprogramming and could define the boundary between protective repair and pathological plasticity.

## 5. Conclusions

Esophageal SMGs are integral to mucosal defense and repair, yet chronic injury can remodel the same compartment toward duct-like, metaplastic, or occasionally neoplastic phenotypes. The mapped evidence supports SMG participation in reflux-associated repair and Barrett biology, but does not establish an exclusive SMG origin of BE or EAC. The most consequential gaps are the lack of longitudinal human evidence, source-resolved lineage information, standardized reporting of glandular phenotypes, and human models that reproduce reflux exposure and the stromal niche.

## Figures and Tables

**Figure 1:**
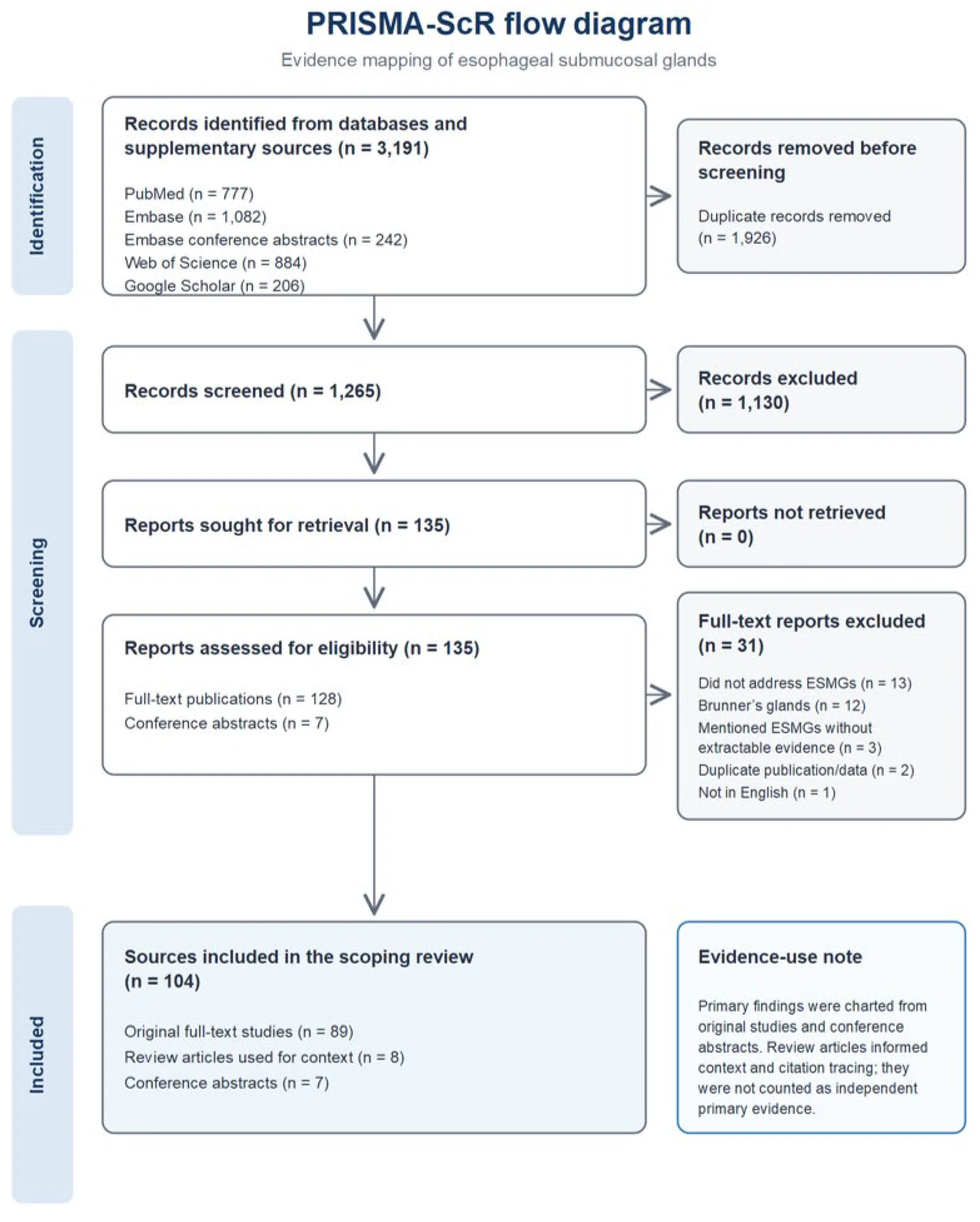
PRISMA-ScR flow diagram of source identification, screening, eligibility assessment, and inclusion. Conference abstracts are displayed separately from full-text publications.

**Figure 2:**
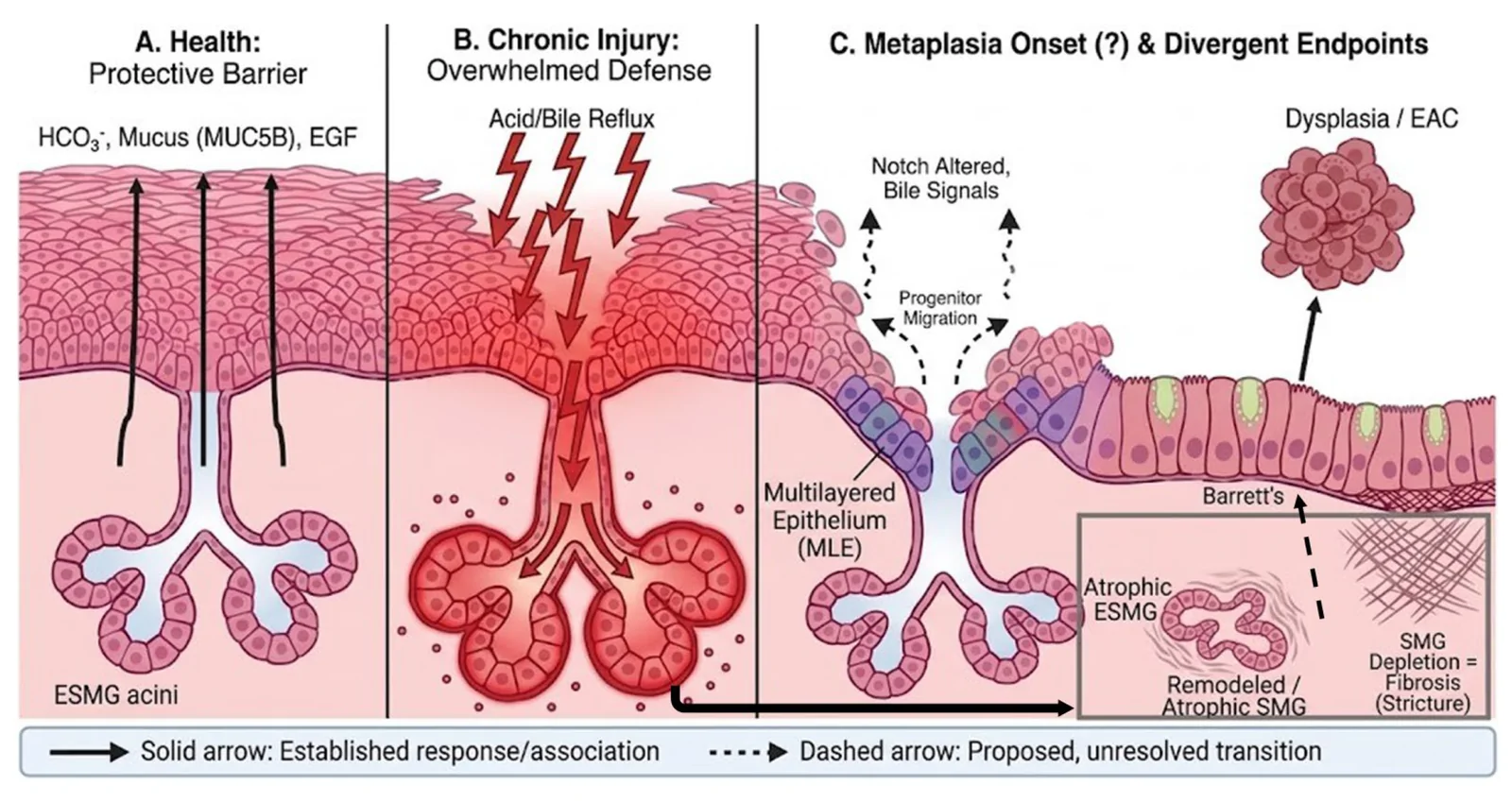
Evidence-informed conceptual continuum of SMG protection, injury, repair, remodeling, and neoplastic association.

**Table 1:** Comparative features and uses of human and animal models in esophageal SMG research.

Species/Model	Epithelial Lining	SMG Status	Epithelial Lining	Key Limitations	Representative Sources
Human	Non-keratinized stratified squamous epithelium	SMGs present, regionally enriched in the proximal and distal third areas	Histopathology, secretion, BE/EAC associations, treatment response	Predominantly observational; limited viable tissue and causal experimentation	[3,5,13,14,15,16,17,18,19,20]
Pig	Non-keratinized stratified squamous epithelium	SMGs present, in the proximal area	Secretory physiology, 3D cultures, injury and ablation depth	Cost, limited genetic tools, and uncertain equivalence to human BE	[21,22,23,24,25]
Dog	Non-keratinized stratified squamous epithelium	SMGs present	Surgical reflux, wound repair, and post-ESD fibrosis	Ethical and cost constraints; limited molecular tools	[26,27,28,29]
Opossum	Stratified squamous epithelium	Dense SMG network	Alkaline secretion and lumen-to-surface pH gradients	Limited genetic tools and restricted availability	[30,31,32,33]
Mouse/rat	Keratinized squamous epithelium; esophageal	SMGs absent	Junctional plasticity, developmental perturbation, and genetic studies	Cannot model a gland-derived esophageal lineage directly	[34,35]

**Table 2:** Evidence map of proposed SMG roles across esophageal pathologies.

Pathology	Mapped SMG Observation	Evidence Base	Evidence-Bounded Interpretation	Representative Sources
Reflux injury and esophagitis	Altered bicarbonate, mucin, EGF, and PGE2 secretion; ductal inflammation or dilation	Human physiology and histology; porcine, canine, and opossum models	SMGs contribute to defense and respond to injury; the extent of recovery after acid suppression is uncertain	[21,22,23,28,29,30,31,32,33,42,43,44,45,51,87]
CLE and BE	ADM, OGM, NSMLC, hybrid squamous-columnar phenotypes, duct-associated squamous islands	Human histology and spatial reconstruction; animal injury models	Compatible with glandular participation in repair or metaplasia, but also with field injury and clonal selection	[20,35,52,53,54,60,63,64,65,88]
Clonality and epithelial origin	Shared or discordant alterations among ducts, Barrett glands, and neosquamous islands	Natural lineage marks and laser-capture genetic studies	Supports local clonal relationships in selected cases; does not establish a universal initiating cell	[57,66,67,68,69]
Barrett-associated dysplasia and EAC	Glandular atrophy/remodeling, EphB2-MYC activity, immune and chromatin changes	Human tissue, single-cell and molecular profiling, porcine spheroids	Nominates SMG-associated states as participants or biomarkers; temporal order remains unresolved	[18,52,53,57,59,66,70,71,72,73,74,75,76,77,78]
Rare SMG-associated neoplasms	MEC, duct adenoma, and duct adenocarcinoma with glandular or mixed differentiation	Case series, case reports, and specialist reviews	Supports SMG-associated tumor categories distinct from conventional EAC; incidence and natural history are poorly defined	[11,79,80,81,82,83,84,85,86,89,90,91,92,93,94,95,96,97]
Endoscopic treatment and repair	Ductal extension may escape superficial ablation; surviving glands may proliferate; gland loss may accompany fibrosis	Human pathology and large-animal ablation/ESD models	Treatment depth may influence both residual disease and repair, but no SMG-based clinical algorithm has been validated	[27,44,48,49,90,94,97,98]

## Data Availability

No primary experimental data were generated. The database-specific search strategies, PRISMA-ScR checklist, and source-characteristics chart are provided in the Supplementary Materials.

## Supplementary Materials

The following supporting information can be downloaded at: https://www.mdpi.com/article/10.3390/cells15191727/s1.
