# Supplementary material for: The Dual Roles of Esophageal Submucosal Glands: Protection and Pathogenesis in Benign and Malignant Disease: A Scoping Review
Source: Cells. 2026 Sep 22;15(19):1727. doi: 10.3390/cells15191727 (PMC13664530; doi:10.3390/cells15191727)
Supplement: Supplementary file 1 [file cells-15-01727-s001.zip › cells-4554309-supplementary/cells-4554309_supplementary_material .pdf]

Supplementary Materials: Scoping Review Methods and Source Characteristics

Manuscript ID: cells-4554309

Supplementary Methods: Database-Specific Search Strategies

PubMed (n = 777)

("oesophagus"[All Fields] OR esophag* OR oesophag* OR "columnar lined"*) AND ("submucosal gland"* OR "sub-mucosal gland"* OR "oesophageal gland"* OR "esophageal gland"*)

Embase (n = 1,082)

(esophageal OR esophagus OR oesophagus OR esophag* OR oesophag* OR 'columnar lined' OR esophagitis) AND (((submucosal OR 'sub mucosal') AND gland*) OR 'oesophageal gland*' OR 'esophageal gland*')

Embase conference abstracts (n = 242)

The Embase strategy above with the conference-abstract publication-type limit; coverage 1985-2024.

Web of Science (n = 884)

(esophageal OR esophagus OR oesophagus OR Barrett* OR "columnar lined"* OR esophagitis) AND ("submucosal gland"* OR "oesophageal gland"* OR "esophageal gland"* OR "sub-mucosal gland"*)

Google Scholar (n = 206)

("esophageal submucosal gland"* OR "esophagus submucosal gland"* OR "oesophagus submucosal gland" OR "sub-mucosal gland") AND (Barrett OR "columnar lined esophagus" OR esophagitis OR "oesophageal gland"* OR "esophageal gland"*)

Search end date: 15 January 2025. Conference-abstract coverage ended in 2024. Confirm whether an updated search is required before resubmission.

Supplementary Table S1: Source-level characteristics

The table below was reconstructed from the EndNote with 104 unique records. Population/sample-size text was extracted only when it appeared in the bibliographic abstract.

No.	Author / year	Title and source	Source type	Population / model	Sample-size information	Evidence domain
1	Al Yassin, T. M. (1977)	Fine structure of squamous epitheilum and submucosal glands of human oesophagus. J Anat, 123, Pt 3, 705-21	Journal article	Human	Human biopsy and resection specimens; exact donor/specimen count was not reported in the accessible article text.	Treatment
2	Haggitt, Rodger C (1978)	Adenocarcinoma complicating columnar epithelium-lined (Barrett's) esophagus. American journal of clinical pathology, 70, 1, 1-5	Journal article	Human	Fourteen primary esophageal adenocarcinomas, including 12 arising in Barrett's esophagus.	BE/CLE/metaplasia; Neoplasia
3	Kuwano, H. (1985)	Glandular or mucus-secreting components in squamous cell carcinoma of the esophagus. Cancer, 56, 3, 514-8	Journal article	Human	A review of 195 patients with carcinoma of the es; the esophagus disclosed 41 cases (21.0%) with glandular a; ortions: glandular type (23 cases), cribriform type (11 ca; cases), cribriform type (11 cases), and mucoepidermoid typ	Neoplasia
4	Sabanathan, S. (1985)	Oesophageal intramural pseudodiverticulosis. Thorax, 40, 11, 849-857	Journal article	Human	The distribution was segmental in 57 cases (59%) and diffuse in 40; symptom and was found in 85 cases (88%); 88 cases out of 9; found in 85 cases (88%); 88 cases out of 97 had radiologic	Anatomy/other SMG evidence
5	Hopwood, D. (1986)	Human oesophageal submucosal glands. Their detection mucin, enzyme and secretory protein content. Histochemistry, 86, 1, 107-12	Journal article	Human	Normal human esophageal tissue used for histochemistry/immunohistochemistry; exact donor/specimen count was not reported in the accessible abstract.	Physiology/secretory defense
6	Gillen, P. (1988)	Experimental columnar metaplasia in the canine oesophagus. Br J Surg, 75, 2, 113-5	Journal article	Dog/canine	Twenty-five adult mongrel dogs distributed across five experimental groups.	Physiology/secretory defense; Injury/repair; BE/CLE/metaplasia
7	Robey, S. S. (1988)	Diagnostic value of cytopathology in Barrett esophagus and associated carcinoma. Am J Clin Pathol, 89, 4, 493-8	Journal article	Human	150 esophageal cytology samples with concurrent endoscopic biopsy specimens.	Injury/repair; BE/CLE/metaplasia; Neoplasia; Treatment
8	Hamilton, B. H. (1989)	In vivo alkaline secretion by mammalian esophagus. Gastroenterology, 97, 3, 640-8	Journal article	Human; Opossum; Rabbit	Baseline secretion measurements included 19 rabbits and 13 opossums; experiment-specific subsets were used for stimulation studies.	Physiology/secretory defense; Injury/repair
9	Attwood, S. E. A. (1993)	WHAT FACTORS ARE EVIDENCED BY STUDY OF THE MUCINS OF THE ESOPHAGEAL GLANDS DURING ESOPHAGITIS. 4th OESO World Congress on the Esophageal Mucosa, 1052, 300-302	Conference	Human	Conference proceeding; exact sample size was not reported in the accessible bibliographic record.	Physiology/secretory defense; Injury/repair
10	Singh, S. (1993)	DETERMINANTS OF ESOPHAGEAL ALKALINE PH ENVIRONMENT IN CONTROLS AND PATIENTS WITH GASTROESOPHAGEAL REFLUX DISEASE. Gut, 34, 3, 309-316	Journal article	Human	Acid and alkaline reflux parameters were studied over 2 days in 30 subjects	Physiology/secretory defense; Injury/repair; BE/CLE/metaplasia
11	Takubo, K. (1993)	Adenoma accompanied by superficial squamous cell carcinoma of the esophagus. Cancer, 71, 8, 2435-8	Journal article	Human	Single-patient case report (n = 1).	BE/CLE/metaplasia; Neoplasia
12	Li, H. (1994)	Mechanisms of columnar metaplasia and squamous regeneration in experimental Barrett's esophagus. Surgery, 115, 2, 176-81	Journal article	Dog/canine	Ten dogs underwent the reflux model; six subsequently underwent antireflux treatment and additional follow-up.	Physiology/secretory defense; Injury/repair; BE/CLE/metaplasia; Methods/model
13	Namiot, Z. (1994)	HUMAN ESOPHAGEAL SECRETION - MUCOSAL RESPONSE TO LUMINAL ACID AND PEPSIN. Gastroenterology, 106, 4, 973-981	Journal article	Human	Twenty-one healthy volunteers underwent esophageal perfusion studies.	Physiology/secretory defense; Injury/repair

14	Rourk, R. M. (1994)	DIMINISHED LUMINAL RELEASE OF ESOPHAGEAL EPIDERMAL GROWTH-FACTOR IN PATIENTS WITH REFLUX ESOPHAGITIS. American Journal of Gastroenterology, 89, 8, 1177-1184	Journal article	Human	Fourteen healthy asymptomatic volunteers and 14 patients with endoscopically confirmed esophagitis	Physiology/secretory defense; Injury/repair; Treatment; Methods/model
15	Rockett, J. C. (1995)	Expression of HLA-ABC, HLA-DR and intercellular adhesion molecule-1 in oesophageal carcinoma. J Clin Pathol, 48, 6, 539-44	Journal article	Human	Thirty-seven esophageal carcinomas were analyzed, with additional normal, inflamed and metaplastic comparator tissues.	Neoplasia
16	Rouse, R. V. (1995)	Esophageal submucosal gland duct adenoma. Am J Surg Pathol, 19, 10, 1191-6	Journal article	Human	Single-patient case report (n = 1).	Physiology/secretory defense; BE/CLE/metaplasia; Neoplasia; Treatment
17	Tobe, N. A. (1995)	Systemic factors in esophageal mucosal protection. Digestion, 56, SUPPL. 1, 38-44	Journal article		Not applicable—narrative review; no original study cohort.	Physiology/secretory defense
18	Rossowski, W. J. (1996)	Vagal stimulation of opossum esophageal submucosal gland bicarbonate secretion. Gastroenterology, 110, 4, A244-A244	Conference	Opossum	Opossum physiological experiment; exact animal number was not reported in the accessible conference abstract record.	Physiology/secretory defense
19	Biddlestone, LR (1998)	The histopathology of treated Barrett's esophagus: squamous reepithelialization after acid suppression and laser and photodynamic therapy. The American journal of surgical pathology, 22, 2, 239-245	Journal article	Human	Thirty-one patients: 16 treated with omeprazole, 10 with KTP laser and 5 with photodynamic therapy.	BE/CLE/metaplasia
20	Van Nieuwenhove, Y. (1998)	Gastroesophageal reflux triggers proliferative activity of the submucosal glands in the canine esophagus. Dis Esophagus, 11, 2, 89-93	Journal article	Dog/canine	Twelve dogs: four cardioplasty, four esophagotomy and four unoperated controls.	Injury/repair
21	Long, J. D. (1999)	Esophageal submucosal glands: structure and function. Am J Gastroenterol, 94, 10, 2818-24	Journal article	Human	Not applicable—narrative review; no original study cohort.	Physiology/secretory defense; Injury/repair
22	Abdulnour-Nakhoul, S. (2000)	Lumen-to-surface pH gradients in opossum and rabbit esophagi: role of submucosal glands. Am J Physiol Gastrointest Liver Physiol, 278, 1, G113-20	Journal article	Human; Opossum; Rabbit	Six opossums and five rabbits; one to three tissue preparations were obtained per animal.	Physiology/secretory defense
23	Arul, G. S. (2000)	Mucin gene expression in Barrett's oesophagus: an in situ hybridisation and immunohistochemical study. Gut, 47, 6, 753-61	Journal article	Human	Forty-six human cases: normal esophagus (n = 8), Barrett's esophagus (n = 18), gastric metaplasia (n = 8), high-grade dysplasia (n = 6) and adenocarcinoma (n = 6).	Physiology/secretory defense; BE/CLE/metaplasia; Neoplasia
24	Guillem, P. (2000)	Mucin gene expression and cell differentiation in human normal, premalignant and malignant esophagus. International Journal of Cancer, 88, 6, 856-861	Journal article	Human	squamous cell carcinoma (n = 17), or Barrett's esophagus; gus with adenocarcinoma (n = 23). In situ hybridization; ssues were obtained from 40 patients undergoing esophagectomy	Physiology/secretory defense; BE/CLE/metaplasia; Neoplasia
25	Tajima, Y. (2000)	Significance of involvement by squamous cell carcinoma of the ducts of esophageal submucosal glands -: <i>Analysis of 201 surgically resected superficial squamous cell carcinomas</i>. Cancer, 89, 2, 248-254	Journal article	Human	two hundred one surgically resected lesions from 140 patients with SSCCE were examined histopathologically	Neoplasia
26	Glickman, J. N. (2001)	Phenotypic characteristics of a distinctive multilayered epithelium suggests that it is a precursor in the development of Barrett's esophagus. American	Journal article	Human	Esophageal mucosal biopsies containing ME from 17 patients with BE were evaluated morphologically, stained with a variety of mucin histochemical stains	Physiology/secretory defense; BE/CLE/metaplasia

		Journal of Surgical Pathology, 25, 5, 569-578				
27	Mandys, V. (2003)	Different patterns of cytokeratin expression in Barrett's esophagus--what is beyond?. Pathol Res Pract, 199, 9, 581-7	Journal article	Human	Ten adults with Barrett's esophagus; gastric mucosal specimens from nine of these patients were also analyzed.	BE/CLE/metaplasia
28	Coad, R. A. (2004)	On the histogenesis of Barrett's oesophagus: A three dimensional study of the relationship between oesophageal gland ducts, Barrett's oesophagus and associated squamous islands. British Journal of Cancer, 91, S55-S55	Conference	Human	Fourteen patients; 46 Barrett's tissue blocks and 15 squamous-island blocks were serially sectioned.	BE/CLE/metaplasia; Neoplasia
29	Daniely, Yaron (2004)	Critical role of p63 in the development of a normal esophageal and tracheobronchial epithelium. American Journal of Physiology-Cell Physiology, 287, 1, C171-C181	Journal article	Human	p63-null and wild-type mouse embryos were compared; the exact number of animals was not stated in the accessible article record.	Anatomy/other SMG evidence
30	Dave, U. (2004)	Distribution of laminins in the basement membranes of the upper gastrointestinal tract and Barrett's oesophagus. J Pathol, 202, 3, 299-304	Journal article	Human; Cell/organoid	Fifteen patients with Barrett's esophagus and five endoscopically normal controls; additional upper-GI specimens were analyzed by site.	BE/CLE/metaplasia; Neoplasia; Treatment
31	Hayashi, Yoshinori (2004)	Submucosal adenoma of the esophagus: case report and review of the literature. Esophagus, 1, 99-102	Review/contextual source	Human	Not applicable	Neoplasia
32	Nakhoul, H. N. (2004)	Immunolocalization of a Na+(HCO3-)n cotransporter in esophageal submucosal glands. Gastroenterology, 126, 4, A542-A543	Conference	Human	Esophageal tissue immunolocalization study; exact biological sample size was not reported in the accessible conference abstract.	Anatomy/other SMG evidence
33	Abdulnour-Nakhoul, S. (2005)	HCO3- secretion in the esophageal submucosal glands. Am J Physiol Gastrointest Liver Physiol, 288, 4, G736-44	Journal article	Pig/porcine	Basal bicarbonate secretion was measured in 19 pig esophagi; tissues from at least four animals were used per antibody.	Physiology/secretory defense; Injury/repair
34	Rey, J. F. (2005)	Endoscopic detection of submucosal glands in the distal esophagus. Gastroenterology, 128, 4, A234-A234	Conference	Human	Conference abstract describing endoscopic observations; exact patient/procedure count was not reported in the accessible record.	Treatment
35	Takubo, K. (2005)	Islands of squamous epithelium and their surrounding mucosa in columnar-lined esophagus: a pathognomonic feature of Barrett's esophagus?. Hum Pathol, 36, 3, 269-74	Journal article	Human	We examined 23 patients with columnar-lined esop; ucosa in 18 (78%) of the 23 patients. When biopsy specimens w; seen in 12 (67%) of the 18 cases. Because the identificat; case in 12 (52%) of the 23 patients examined in this study	BE/CLE/metaplasia; Treatment
36	White, R. J. (2005)	Morphology and glycoconjugate content of opossum esophageal epithelium and glands: Regional heterogeneity and effects of acid-induced mucosal injury and recovery. Digestive Diseases and Sciences, 50, 9, 1591-1604	Journal article	Opossum	Opossum esophagi were assessed in control, acid-injury and recovery groups; site-specific denominators ranged from 4 to 13.	Injury/repair; Methods/model
37	Abdulnour-Nakhoul, S. M. (2006)	Tegaserod stimulates bicarbonate secretion by esophageal submucosal glands and protects against acid-pepsin injury in a pig model. Gastroenterology, 130, 4, A405-A405	Conference	Pig/porcine	Porcine ex vivo injury experiments; exact biological sample size was not reported in the accessible conference abstract.	Physiology/secretory defense; Injury/repair; Methods/model
38	Paulson, T. G. (2006)	Neosquamous epithelium does not typically arise from Barrett's epithelium. Clin Cancer Res, 12, 6, 1701-6	Journal article	Human	Barrett's epithelium from 20 patients by microdissection and e; helium. RESULTS: In 1 of 20 patients, a focus of NSE had a 14; The NSE in the	BE/CLE/metaplasia; Neoplasia; Treatment

					remaining 19 patients was wild-type for p16 or p16 or TP53.	
39	Abdulnour-Nakhoul, S. (2007)	Characterization of esophageal submucosal glands in pig tissue and cultures. Dig Dis Sci, 52, 11, 3054-65	Journal article	Human; Pig/porcine; Cell/organoid	Porcine esophageal tissues and primary gland cultures; no single overall animal/culture sample size was reported.	Physiology/secretory defense; BE/CLE/metaplasia; Methods/model
40	Majewski, M. (2007)	GENDER-RELATED DIFFERENCE IN COMPOSITION OF SECRETION FROM ESOPHAGEAL SUBMUCOSAL MUCOUS GLANDS, IN RESPONSE TO STIMULATION OF SEROTONERGIC PATHWAY, IN PATIENTS WITH GASTROESOPHAGEAL REFLUX DISEASE. American Journal of Gastroenterology, 102, S152-S153	Conference	Human	Thirty-eight patients with GERD (26 female and 12 male) in a double-blind crossover study.	Physiology/secretory defense; Injury/repair
41	Abdulnour-Nakhoul, S. (2008)	The effect of tegaserod on esophageal submucosal glands bicarbonate and mucin secretion. Dig Dis Sci, 53, 9, 2366-72	Journal article	Human; Pig/porcine; Rabbit	Isolated pig and rabbit esophagi; experiment-specific denominators were generally 5-7 tissue preparations.	Physiology/secretory defense; Injury/repair
42	Leedham, S. J. (2008)	Individual crypt genetic heterogeneity and the origin of metaplastic glandular epithelium in human Barrett's oesophagus. Gut, 57, 8, 1041-8	Journal article	Human	Six biopsies from five patients and 10 esophagectomy blocks from three patients; 164 Barrett's crypts and 21 squamous islands were analyzed.	BE/CLE/metaplasia; Neoplasia; Methods/model
43	Wang, S. W. (2008)	Early adenocarcinoma originating in submucosal gland of thoracic esophagus presenting as submucosal tumor. Endoscopy, 40, E237-E238	Journal article	Human	Single-patient case report (n = 1).	Neoplasia
44	Abdulnour-Nakhoul, S. (2009)	IDENTIFICATION OF CFTR AND SLC26A6 IN THE ESOPHAGEAL SUBMUCOSAL GLANDS. Journal of Investigative Medicine, 57, 1, 375-375	Journal article	pig	Porcine esophageal tissue/cell preparations; exact biological sample size was not reported in the accessible abstract.	Anatomy/other SMG evidence
45	Peters, J. H. (2010)	The molecular pathogenesis of Barrett's esophagus: common signaling pathways in embryogenesis metaplasia and neoplasia. J Gastrointest Surg, 14 Suppl 1, S81-7	Conference		Not applicable—narrative review; no original study cohort.	Injury/repair; BE/CLE/metaplasia
46	Segat, D. (2010)	Pericentriolar material analyses in normal esophageal mucosa, Barrett's metaplasia and adenocarcinoma. Histol Histopathol, 25, 5, 551-60	Journal article	Human	Twenty-eight esophagectomy cases were examined.	Injury/repair; BE/CLE/metaplasia; Neoplasia
47	Wang, D. H. (2010)	Aberrant epithelial-mesenchymal Hedgehog signaling characterizes Barrett's metaplasia. Gastroenterology, 138, 5, 1810-22	Journal article	Human; Mouse/murine; Cell/organoid	Tissue microarrays represented 96 esophagectomy cases; paired frozen Barrett's/squamous samples from 15 cases were used for qRT-PCR.	Injury/repair; BE/CLE/metaplasia; Neoplasia; Methods/model
48	Abdulnour-Nakhoul, S. (2011)	Ion transport mechanisms linked to bicarbonate secretion in the esophageal submucosal glands. Am J Physiol Regul Integr Comp Physiol, 301, 1, R83-96	Journal article	Human	Porcine esophagi and isolated gland preparations; at least four animals were used per immunolabeling experiment, with assay-specific denominators.	Physiology/secretory defense
49	Lorinc, E. (2012)	Submucosal glands in the columnar-lined oesophagus: evidence of an association with metaplasia and neosquamous epithelium. Histopathology, 61, 1, 53-8	Journal article	Human	Seven patients provided seven esophageal resection specimens; 300 tissue pieces were evaluated.	BE/CLE/metaplasia; Treatment
50	Nicholson, A. M. (2012)	Barrett's metaplasia glands are	Journal article	Human; Cell/organoid	Eleven patients contributed Barrett's	BE/CLE/metaplasia; Neoplasia;

		clonal, contain multiple stem cells and share a common squamous progenitor. Gut, 61, 10, 1380-9			specimens; cytochrome-c-oxidase-deficient glands were identified in seven, with sequencing in four.	Methods/model
51	Zeki, S. S. (2013)	Clonal selection and persistence in dysplastic Barrett's esophagus and intramucosal cancers after failed radiofrequency ablation. Am J Gastroenterol, 108, 10, 1584-92	Journal article	Human	longitudinal case series of 19 patients with BE and high-grade dysplasia (HGD).	BE/CLE/metaplasia; Neoplasia; Treatment
52	Braxton, D. R. (2014)	Necrotizing sialometaplasia-like change of the esophageal submucosal glands is associated with Barrett's esophagus. Virchows Archiv, 465, 2, 135-143	Journal article	Human	Eighty-two esophagectomy cases (45 Barrett's and 37 non-Barrett controls), plus 25 endoscopic mucosal-resection cases.	Physiology/secretory defense; Injury/repair; BE/CLE/metaplasia; Neoplasia; Treatment; Methods/model
53	Hayes, S. J. (2014)	Immunohistochemical assessment of NY-ESO-1 expression in esophageal adenocarcinoma resection specimens. World J Gastroenterol, 20, 14, 4011-6	Journal article	Human	: Four out of a total of 27 cases of esophageal adenocarcinoma	BE/CLE/metaplasia; Neoplasia; Treatment
54	Jansen, M. (2014)	Is it justified to ablate flat-type esophageal squamous cancer? An analysis of endoscopic submucosal dissection specimens of lesions meeting the selection criteria of radio frequency studies. Gastrointestinal Endoscopy, 80, 6, 995-1002	Journal article	Human	. Six of the 17 specimens (35%) showed relevant differences	Neoplasia; Treatment
55	Vega, M. E. (2014)	Inhibition of Notch signaling enhances transdifferentiation of the esophageal squamous epithelium towards a Barrett's-like metaplasia via KLF4. Cell Cycle, 13, 24, 3857-66	Journal article	Human; Cell/organoid	Seven paired Barrett's and adjacent normal squamous tissue samples were used for expression profiling; complementary cell-line experiments were also performed.	Physiology/secretory defense; BE/CLE/metaplasia; Neoplasia; Methods/model
56	Garman, K. S. (2015)	Ductal metaplasia in oesophageal submucosal glands is associated with inflammation and oesophageal adenocarcinoma. Histopathology, 67, 6, 771-82	Journal article	Human	343 unique ESMGs from 30 control patients, 24 patients with treatment-naïve high-grade columnar dysplasia (HGD) or EAC, and 23 non-EAC esophagectomy cases.	BE/CLE/metaplasia; Neoplasia; Methods/model
57	Lorinc, E. (2015)	The immunophenotypic relationship between the submucosal gland unit, columnar metaplasia and squamous islands in the columnar-lined oesophagus. Histopathology, 67, 6, 792-8	Journal article	Human	Eight tissue blocks from three esophagectomy patients.	BE/CLE/metaplasia; Neoplasia; Treatment
58	Lorinc, E. (2015)	Hyperplasia of the submucosal glands of the columnar-lined oesophagus. Histopathology, 66, 5, 726-31	Journal article	Human	Eight resection specimens (seven columnar-lined esophagus cases and one non-CLO control), containing 339 ESMGs.	Injury/repair; BE/CLE/metaplasia; Treatment
59	Kouznetsova, I. (2007)	Localization of TFF3 peptide in human esophageal submucosal glands and gastric cardia: differentiation of two types of gastric pit cells along the rostro-caudal axis. Cell Tissue Res, . 328(2): p. 365-74.	Journal article	Human	Biopsy specimens<br>12 (BM1–BM12)<br>Z+2, Z, Z-0.5, Z-4, corpus<br><br>Biopsy specimens<br>8 (B21–B28)<br>Z+2, Z, Z-1, corpus<br><br>Surgical specimens<br>9 (M34, M46, M47, M48, M63, M74, M82, M90, M94)<br>Esophagus, cardia<br><br>Post-mortem specimens<br>5 (P69/05, P70/05, P72/05, P113/05, P131/05)	BE/CLE/metaplasia; Neoplasia

					Z+2	
60	McDonald, S. A. (2015)	The Barrett's Gland in Phenotype Space. Cell Mol Gastroenterol Hepatol, 1, 1, 41-54	Journal article		Not applicable—review/commentary; no original study cohort.	BE/CLE/metaplasia; Neoplasia
61	McDonald, S. A. C. (2015)	OPINION Barrett oesophagus: lessons on its origins from the lesion itself. Nature Reviews Gastroenterology & Hepatology, 12, 1, 50-60	Review/contextual source		Not applicable	BE/CLE/metaplasia; Methods/model
62	Yorita, K. (2015)	A Novel Case of an Unusual Esophageal Submucosal Tumor: An Esophageal Submucosal Gland Duct Hamartoma (vol 110, pg 1261, 2015). American Journal of Gastroenterology, 110, 11, 1634-1634	Journal article	Human	Single-patient case report (n = 1); this citation is an erratum/correction to the original report.	Neoplasia
63	Lavery, D. L. (2016)	Evolution of oesophageal adenocarcinoma from metaplastic columnar epithelium without goblet cells in Barrett's oesophagus. Gut, 65, 6, 907-U208	Journal article	Human	Single-patient esophagectomy case (n = 1).	BE/CLE/metaplasia; Neoplasia
64	Nie, L (2016)	Esophageal submucosal gland duct adenoma: a clinicopathological and immunohistochemical study with a review of the literature. Diseases of the Esophagus, 29, 8, 1048-1053	Review/contextual source	Human	Not applicable	Neoplasia
65	Sarosiek, J. (2016)	Does the healing of the esophageal mucosa improve the function of the esophageal submucosal and salivary glands?. Ann N Y Acad Sci, 1380, 1, 155-161	Journal article	Human	Not applicable—narrative review; no original study cohort.	Physiology/secretory defense; Injury/repair
66	Soucy, G. (2016)	Histologic Features Associated With Columnar-lined Esophagus in Distal Esophageal and Gastroesophageal Junction (GEJ) Biopsies From GERD Patients: A Community-based Population Study. American Journal of Surgical Pathology, 40, 6, 827-835	Journal article	Human	biopsies of the SCJ from 544 patients in a large prospective c	Injury/repair; BE/CLE/metaplasia; Treatment
67	Tripathi, Monika (2016)	Rare tumors of esophageal squamous mucosa. Annals of the New York Academy of Sciences, 1381, 1, 122-132	Journal article	Human	Not applicable—narrative review; no original study cohort.	Neoplasia
68	Tsutsumi, S. (2016)	Distant lymph node metastases caused by esophageal cancer invasion to the lamina propria: a case report. Surg Case Rep, 2, 1, 143	Journal article	Human	Single-patient case report (n = 1).	Neoplasia; Treatment
69	Fass, Ofer (2017)	Bile Acid-Mediated Transdifferentiation of Stem Cells From the Deep Esophageal Glands.	Thesis/dissertation	Human	Not applicable as a single cohort—doctoral thesis comprising multiple experiments with experiment-specific sample sizes.	Anatomy/other SMG evidence
70	Garman, K. S. (2017)	Origin of Barrett's Epithelium: Esophageal Submucosal Glands. Cell Mol Gastroenterol Hepatol, 4, 1, 153-156	Journal article	Human	Not applicable—review/commentary; no original study cohort.	Injury/repair; BE/CLE/metaplasia; Neoplasia; Methods/model
71	Honda, M. (2017)	The mechanism of esophageal stricture after endoscopic resection: Histological and biomechanical evaluation in a canine model. Annals of Cancer Research and Therapy, 25, 1, 30-37	Journal article	Human; Dog/canine	Nine beagle dogs underwent circumferential endoscopic submucosal dissection.	Treatment; Methods/model

72	Kruger, L. (2017)	Ductular and proliferative response of esophageal submucosal glands in a porcine model of esophageal injury and repair. Am J Physiol Gastrointest Liver Physiol, 313, 3, G180-G191	Journal article	Human; Pig/porcine	Thirty pigs: three at each of seven recovery intervals, six additional day-7 animals and three sham controls.	Injury/repair; BE/CLE/metaplasia; Neoplasia; Treatment; Methods/model
73	von Furstenberg, Richard J (2017)	Porcine esophageal submucosal gland culture model shows capacity for proliferation and differentiation. Cellular and molecular gastroenterology and hepatology, 4, 3, 385-404	Journal article	Pig/porcine; Cell/organoid	Human IHC: seven cases; porcine cultures: three independent animals; in vivo EdU comparison: two pigs.	Methods/model
74	Bartel, M. J. (2018)	Subsquamous intestinal metaplasia is common in treatment-naive Barrett's esophagus. Gastrointestinal Endoscopy, 87, 1, 67-74	Journal article	Human	One hundred six treatment-naive Barrett's patients, providing 515 squamous biopsy specimens.	Injury/repair; BE/CLE/metaplasia; Neoplasia
75	Bartel, M. J. (2018)	Esophageal submucosal glands as a potential source of subsquamous intestinal metaplasia in Barrett's esophagus Response. Gastrointestinal Endoscopy, 88, 1, 201-201	Journal article	Human	Not applicable—response letter with no new study cohort.	BE/CLE/metaplasia
76	Martinez-Uribe, O. (2018)	INCREASED GASTRIN RESPONSIVENESS AFTER INJURY IN ESOPHAGEAL SUBMUCOSAL GLANDS. Gastroenterology, 154, 6, S207-S208	Conference	Human	Human archival specimens, an in vivo porcine injury model and 3D ESMG cultures; exact numbers were not reported in the conference abstract.	Injury/repair
77	Owen, Richard Peter (2018)	Single cell RNA-seq reveals profound transcriptional similarity between Barrett's oesophagus and oesophageal submucosal glands. Nature communications, 9, 1, 4261	Journal article	Human	Bulk profiling used 13 Barrett's patients; single-cell RNA-seq included eight patients (six Barrett's, two non-Barrett controls) and 4,237 cells.	BE/CLE/metaplasia; Methods/model
78	Wong, M. C. S. (2018)	Global Incidence and mortality of oesophageal cancer and their correlation with socioeconomic indicators temporal patterns and trends in 41 countries. Scientific Reports, 8	Journal article	Human	Population-level incidence and mortality data from 41 countries; no single individual-patient cohort.	Neoplasia
79	Zhang, X. (2018)	The microscopic anatomy of the esophagus including the individual layers, specialized tissues, and unique components and their responses to injury. Ann N Y Acad Sci, 1434, 1, 304-318	Journal article	Human	Not applicable—narrative anatomy review; no original study cohort.	Physiology/secretory defense; Injury/repair
80	Jules Lin (2019)	Chapter 4 - Relevant Anatomic Relations of the Esophagus. Shackelford's Surgery of the Alimentary Tract, 2 Volume Set (Eighth Edition), 1, 31-43	Journal article	Human	Not applicable—textbook chapter; no original study cohort.	Anatomy/other SMG evidence
81	Que, J. (2019)	Pathogenesis and Cells of Origin of Barrett's Esophagus. Gastroenterology, 157, 2, 349-364 e1	Review/contextual source	Human	Not applicable	Injury/repair; BE/CLE/metaplasia; Neoplasia
82	Saxena, A. K. (2019)	Comparison of esophageal submucosal glands in experimental models for esophagus tissue engineering applications. Esophagus, 16, 1, 77-84	Journal article	Human; Pig/porcine; Mouse/murine	Five esophageal specimens were examined for each of five animal model species.	Physiology/secretory defense; Methods/model
83	Straub, Danielle (2019)	Glyco-conjugated bile acids drive the initial metaplastic gland formation from multi-layered glands through crypt-	Journal article	Mouse/murine	Multiple mouse cohorts: reflux surgery n = 4/time point; patient-refluxate exposure n = 50; individual bile acids n = 6/group; lineage tracing n = 5-	Methods/model

		fission in a murine model. PLoS One, 14, 7, e0220050			10/group.	
84	Johncilla, M. (2020)	Role of submucosal glands in the development and progression of carcinoma in Barrett's oesophagus. Pathology, 52, 3, 310-317	Journal article	Human	Not applicable—narrative review; no original study cohort.	Physiology/secretory defense; Injury/repair; BE/CLE/metaplasia; Neoplasia; Treatment
85	Korbut, E. (2020)	Molecular Profile of Barrett's Esophagus and Gastroesophageal Reflux Disease in the Development of Translational Physiological and Pharmacological Studies. Int J Mol Sci, 21, 17	Review/contextual source	Human; Cell/organoid	Not applicable	Physiology/secretory defense; Injury/repair; BE/CLE/metaplasia; Neoplasia; Methods/model
86	Kruger, L. (2020)	Lubiprostone protects esophageal mucosa from acid injury in porcine esophagus. Am J Physiol Gastrointest Liver Physiol, 318, 4, G613-G623	Journal article	Human; Pig/porcine	Ex vivo porcine esophageal preparations with experiment-specific denominators, generally n = 4-6 per condition; no single total animal count was reported.	Physiology/secretory defense; Injury/repair; BE/CLE/metaplasia; Neoplasia; Methods/model
87	Nie, L. (2020)	Submucosal gland neoplasms of the esophagus: an update and review. Esophagus, 17, 4, 376-384	Review/contextual source	Human	Not applicable	Neoplasia
88	Othman, M. O. (2020)	Endoscopic findings of esophageal gland duct adenoma resected by endoscopic submucosal dissection Commentary. Gastrointestinal Endoscopy, 92, 4, 962-962	Journal article	Human	Not applicable—commentary on a single published case; no new study cohort.	Neoplasia; Treatment
89	Wang, W. L. (2020)	Risk factors and pathological characteristics for intraductal tumor spread of submucosal gland in early esophageal squamous cell neoplasia. Sci Rep, 10, 1, 6860	Journal article	Human	One hundred sixty early esophageal squamous neoplasia lesions treated by endoscopic submucosal dissection.	Neoplasia; Treatment
90	Janmaat, Vincent T (2021)	HOXA13 in etiology and oncogenic potential of Barrett's esophagus. Nature Communications, 12, 1, 3354	Journal article	Human	Human tissue cohorts included healthy esophagus (n = 12), GIP (n = 5), CLE (n = 14), BE (n = 13), EAC (n = 12) and comparison GI tissues (n = 6-14/group).	BE/CLE/metaplasia
91	Nowicki-Osuch, Karol (2021)	Molecular phenotyping reveals the identity of Barrett's esophagus and its malignant transition. Science, 373, 6556, 760-767	Journal article	Human	Single-cell atlas: 43,000 cells from 43 samples across seven sites from 14 donors/patients; ESMGs were profiled from three disease-free donors.	BE/CLE/metaplasia
92	Overwater, A. (2021)	Extension of early esophageal squamous cell neoplasia into ducts and submucosal glands and the role of endoscopic ablation therapy. Gastrointestinal Endoscopy, 94, 4, 832-842.e2	Journal article	Human; Pig/porcine	ESCN from a Japanese (n = 65) and Dutch cohort (n = 1; = 65) and Dutch cohort (n = 14) were evaluated for pres; cumferential RFA (c-RFA; n = 4), focal CBA (n = 20), an; -RFA; n = 4), focal CBA (n = 20), and focal RFA (n = 3)	Treatment; Methods/model
93	Saez de Gordoa, K. (2021)	Submucosal gland adenocarcinoma of the esophagus. A rare non-Barrett's associated tumor. Gastroenterol Hepatol, 44, 5, 367-369	Journal article	Human	Single-patient case report (n = 1).	BE/CLE/metaplasia; Neoplasia
94	Venkitachalam, Srividya (2021)	The EphB2-MYC Axis is a Major Determinant of Barrett's Pathobiology and a Therapeutic Vulnerability in Esophageal Cancer. bioRxiv, 2021.05. 13.444044	Journal article	Human	Discovery tissue cohort n = 89; independent validation cohort n = 885 across normal squamous, Barrett's, dysplasia, EAC and gastric tissues.	BE/CLE/metaplasia; Neoplasia
95	Wang, Hui (2021)	Roles of Cl-/HCO 3-anion exchanger 2 in the physiology and pathophysiology of the digestive system. Molecular Medicine Reports, 24, 1, 1-11	Journal article	Human	Not applicable—narrative review; no original study cohort.	Anatomy/other SMG evidence
96	Zhu, Zhu (2021)	Whole-Exome Sequencing of	Journal article	Human	Single esophageal submucosal gland	Neoplasia

		Esophageal Submucosal Gland Duct Adenocarcinoma.			duct adenocarcinoma case (n = 1) analyzed by whole-exome sequencing.	
97	Evans, J. A. (2022)	Clonal Transitions and Phenotypic Evolution in Barrett's Esophagus. Gastroenterology, 162, 4, 1197-1209 e13	Journal article	Human	Four clinical cohorts: 64 biopsies from 51 patients; 20 patients; 167 samples from 49 patients; and 31 biopsies from 19 post-esophagectomy patients.	BE/CLE/metaplasia; Neoplasia
98	Konda, V. (2022)	An Endoscopic and Histologic Study on Healing of Radiofrequency Ablation Wounds in Patients With Barrett's Esophagus. Am J Gastroenterol, 117, 10, 1583-1592	Journal article	Human	the squamous islands. In 2 patients, biopsies of such island; weeks after RFA in 6 of 7 patients. DISCUSSION: RFA wounds	Injury/repair; BE/CLE/metaplasia; Treatment
99	Venkitachalam, Srividya (2022)	The Ephrin B2 receptor tyrosine kinase is a regulator of proto-oncogene MYC and molecular programs central to barrett's Neoplasia. Gastroenterology, 163, 5, 1228-1241	Journal article	Human	Discovery tissue cohort n = 89; independent validation cohort n = 885 across normal squamous, Barrett's, dysplasia, EAC and gastric tissues.	BE/CLE/metaplasia
100	Chen, S. Y. (2023)	Modified endoscopic submucosal tunnel dissection for large esophageal submucosal gland duct adenoma: A case report. World J Gastrointest Surg, 15, 5, 1000-1006	Journal article	Human	Single-patient case report (n = 1).	Neoplasia; Treatment
101	Garman, Katherine S (2023)	Genetic Defect in Submucosal Gland–Associated Caveolin-3: A New Paradigm in Esophageal Adenocarcinoma Risk. Gastroenterology, 165, 6, 1561-1564. e3	Journal article	Human	Not applicable—commentary/perspective; no original study cohort.	Neoplasia
102	Nowicki-Osuch, K. (2023)	Single-Cell RNA Sequencing Unifies Developmental Programs of Esophageal and Gastric Intestinal Metaplasia. Cancer Discovery, 13, 6, 1346-1363	Journal article	Human	Integrated single-cell atlas of more than 146,000 cells from multiple current and published gastrointestinal cohorts; cohort-level n is reported in Supplementary Table S1.	BE/CLE/metaplasia; Neoplasia; Methods/model
103	Maurer, J. (2024)	An Incidental Case of Esophageal Submucosal Gland Duct Adenoma. American Journal of Gastroenterology, 119, 10S, S2289-S2290	Conference	Human	Single-patient conference case report (n = 1).	Neoplasia
104	Nie, L. (2024)	Submucosal gland differentiation and implications in esophageal basaloid squamous cell carcinomas. Histology and Histopathology, 39, 3, 399-409	Journal article	Human	One hundred fifty-two esophageal basaloid squamous cell carcinomas.	Neoplasia
